# Development and validation of a questionnaire to assess the doctors and nurses knowledge of acute oxygen therapy

**DOI:** 10.1371/journal.pone.0211198

**Published:** 2019-02-04

**Authors:** Olufemi O. Desalu, Adeniyi O. Aladesanmi, Olutobi B. Ojuawo, Christopher M. Opeyemi, Rasheedah M. Ibraheem, Zakari A. Suleiman, Olanrewaju O. Oyedepo, Kikelomo T. Adesina, Taofeek Oloyede, Emmanuel O. Sanya, Oxygen Study Group

**Affiliations:** 1 Department of Medicine, University of Ilorin Teaching Hospital, Ilorin, Nigeria; 2 Department of Child Health, University of Ilorin Teaching Hospital, Ilorin, Nigeria; 3 Department of Anaesthesia, University of Ilorin Teaching Hospital, Ilorin, Nigeria; 4 Department of Obstetrics and Gynaecology, University of Ilorin Teaching Hospital, Ilorin, Nigeria; University of California San Diego, UNITED STATES

## Abstract

**Background:**

Prescription and administration of oxygen in emergencies by healthcare providers are reported to be inappropriate in most settings. There is a huge gap in the knowledge of health care providers on various aspects of oxygen therapy, and this may be a barrier to optimal oxygen administration. Hence, it is essential to ascertain providers’ knowledge of acute oxygen therapy so that appropriate educational interventions are instituted for better delivery. There is no available validated instrument to assess knowledge of acute oxygen therapy. The study aimed to develop, validate and evaluate the test-retest reliability of a questionnaire to determine the doctors and nurses understanding of acute oxygen therapy.

**Methods:**

This study involved the development of the questionnaire contents by a literature review, assessment of face validity (n = 5), content validity, using a panel of experts (n = 10), item analysis and test-retest reliability among a sample (n = 121) of doctors and nurses.

**Results:**

Face validity indicated that the questionnaire was quick to complete (10–15 min), most items were easy to follow and comprehensible. The global content validity index (S-CVI) was 0.85. The test-retest reliability statistics showed a kappa coefficient of 0.546–0.897 (all P<0.001) and percentage agreement of 80–98.3% indicating high temporal stability in the target population. In total, 90% of the items fulfilled the reliability acceptance criteria. Item discrimination analysis showed that most questions were at an acceptable level. The final questionnaire included 37 item questions and eight sections.

**Conclusion:**

The designed questionnaire is a reliable and valid tool for assessing knowledge of acute oxygen therapy among doctors and nurses.

## Introduction

Oxygen is a commonly used medication in the clinical setting [1]. Low oxygen level in the blood can result in cellular dysfunction, organ failure, and death. The gas is like any other medical drug, and when used appropriately it can reduce mortality, and when administered wrongly it may be harmful to the recipient and resulting in adverse consequences [2]. It was erroneously and previously believed that too much oxygen would not hurt [3]. However, recent clinical data from systematic reviews and randomized clinical trial on the use of acute oxygen therapy in patients with stroke and acute myocardial infarction have disapproved this notion [4–6]. Excessive administration of oxygen can be harmful, especially in chronic obstructive pulmonary disease (COPD) patients with hypercapnic respiratory failure and those with other pulmonary conditions associated with hypercapnic respiratory failure [7]. The inappropriate administration also leads to increased hospital lengths of stay, higher rates of admission to high dependency units, and an increased risk of death [8–10]. Uncontrolled oxygen administration, mainly when delivered at high concentrations, can result in a worsening of hypercapnia which is primarily caused by hypoxic vasoconstriction [11] and not due to reduced hypoxic drive as previously believed [12]. As a result of these documented harmful effects of out of controlled oxygen administration, the use of titrated oxygen therapy in the vulnerable patient group has been recommended for many years by safety agency and several international respiratory societies [7, 13–16]. For safety reason, oxygen should be treated like any other prescription drug with the orders for therapy included in a treatment (medication) chart before administration. The prescription must include specification of dose, methods of delivery, therapy duration and monitoring or define a target arterial oxygen saturation range (SatO_2_) [7, 13–17]. Many previous studies worldwide [18–26] and unpublished data in our setting have reported that the prescription and administrations of emergency oxygen by healthcare providers are inappropriate and inadequate. Hence, it is essential to ascertain healthcare providers’ knowledge on the use of oxygen for acute respiratory insufficiency, so that the appropriate educational interventions can be tailored to the task. To the best of authors’ knowledge, there is no validated instrument for assessing the knowledge of doctors and nurses on the appropriate administration of oxygen in emergencies. We hypothesized that a 14-day test-retest analysis of the responses of health care providers to the questionnaire would demonstrate the validity and reliability of the questionnaire. This study aimed to develop, validate and assess the test-retest reliability of a self-administered questionnaire to evaluate the doctors and nurses knowledge of acute administration of oxygen in our setting.

## Materials and methods

### Study population

The study population consisted of physicians and nurses working in secondary and tertiary hospitals in Ilorin city and two neighbouring towns, in Nigeria.

### Development of the questionnaire

**Fig 1:**
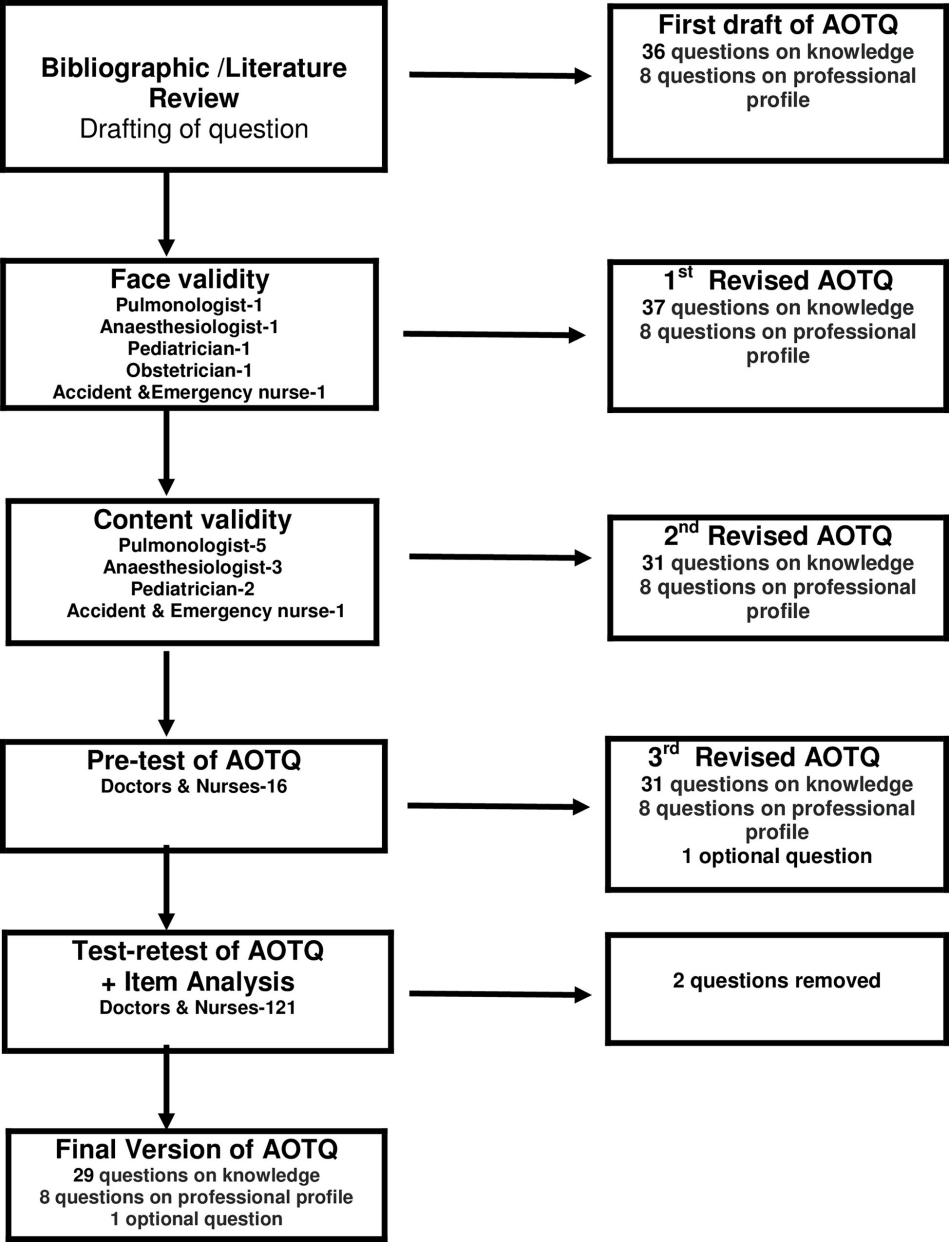
Flow chart of the development and validity of the questionnaire.

The development of the questionnaire was conducted in five significant steps. A flow chart illustrating the development and validity process of the knowledge of acute oxygen therapy questionnaire (AOTQ) is presented in Fig 1. We performed an extensive literature review to identify existing questionnaires that evaluated the knowledge and practice of acute oxygen therapy, an audit of emergency oxygen therapy and published guidelines of oxygen therapy. The questionnaire was developed from previous studies [1, 2, 14–24] on acute oxygen therapy. The individual questions with items were not related to each other, and therefore exploratory factor analysis was not applicable.

### Face validity

Face validity involves the scrutiny of all the items in the questionnaire to establish that they are a valid measure of the concept. It evaluates the appearance of the questionnaire regarding feasibility, readability, consistency of style and formatting, and the clarity of the language used [27, 28]. The initial version of the questionnaire was drafted by the pulmonologist and the questionnaire items, and content was developed in collaboration with four other investigators: anaesthesiologist, pediatric pulmonologist, an obstetrician and emergency nurse who administer oxygen to patients on a regular basis. In determining the face validity of the questionnaire, we used an evaluation form designed to assist respondents in assessing each question items. The items were evaluated for 1) the clarity 2) easy comprehension, 3) the layout and style and 4) whether the items effectively capture the subject being assessed and 5) to provide suggestions to be incorporated in the next version of the questionnaire.

### Content validity

The content validity of an instrument is typically achieved by a thorough analysis of the instrument by experts familiar with the construct of interest or experts on the research subject [27–29]. Ten specialists in the field of medical science were identified and invited to review the instrument for content validity. They consisted of four pulmonologists, three anaesthesiologists, and two pediatricians and an accident and emergency nurse tutor. The inclusion criteria for selecting panel for content validity were 1) licensed practitioners (>10 years) who 2) engaged in teaching medical and nursing students and use the recommendations about oxygen therapy [30] and 3) availability to complete the task within the specified time frame. The instruments were distributed via the email or directly to them in a postal envelope with an introductory cover letter. On completion of the review, the completed instruments were returned to the author through the same medium. The panelists were provided with detailed instruction to identify the correlation between items of the instrument and the aforementioned thematic sections. The panel of raters reviewed the questionnaire for readability, clarity, and comprehensiveness and came to some level of agreement as to which question should be retained in the final questionnaire. The rating was dichotomous; an item was rated ‘favorable' (assigned a score of +1) or ‘unfavorable' (assigned score of +0). Furthermore, the panels of experts were also instructed to identify deficient areas and provide suggestions on ways to ensure clarity and brevity based on difficulties encountered in deciphering the instructions for filling out the instrument [31]. The cumulated level of agreement among the experts was averaged and assigned a numerical value known as the content validity index (CVI) [30–31]. We used the content validity of individual items (I-CVI) and the content validity of the overall scale (S-CVI) to assess content validity. The scale level content validity indices (S‑CVI) was calculated from I‑CVI. [28–30]. Politis, et al. recommended an I-CVI of 0.78 for 6 to 10 experts [29].

### Pilot study and questionnaire revision

A preliminary version was pilot-tested on a convenience sample of 16 consenting doctors and nurses (6 nurses and ten doctors including an emergency physician). The questionnaire took approximately 10–15 min to complete, and they provided feedback on any misleading and confusing question items. The suggestions from the participants included reformulating and rewording of some items and removing potentially repetitive ones. All reported feedback was sent to the lead investigator for necessary actions. Questions were removed or modified based on these suggestions.

### Test re-test reliability

Test-retest correlation gives an indication of stability over time [28]. We conducted a test-retest study using a convenience sample of 121 doctors and nurses drawn from all the public and private hospitals. The questionnaires were delivered together with a cover letter, outlining the study objectives, confidentiality and highlighting the importance of their participation. The questionnaires were distributed to each participant twice, at an interval of two to three weeks; assess reliability in line with the scientific literature [32]. Participants were contacted for the retest and prompted to complete the same questionnaire on the same day of the week. One of the investigators was sent to the participants seven days after to remind them to increase the response rates.

### Items analysis of AOTQ

We also performed question items analysis by calculating item discrimination (item-total correlations) and item difficulty (% correct) of each knowledge question in AOTQ. These analyses enable us to do a critical evaluation of each question to be retained, revised or rejected [33]. Item difficulty helps us to know how difficult or easy the questions were, while the item discrimination assists in determining whether the questions were able to discriminate between subjects who performed well, from those who did not on the AOTQ [33]. The ideal difficulty levels for multiple-choice items concerning discrimination potential are 77% for three-response multiple-choice and 85% for True-false (two-response multiple-choice)[33]. The items that showed item-to-total correlations < 0.2 were to be rejected or improved by revision [34].

### The final version of AOTQ

Based on the validity and reliability test of the previous steps identified above, we produced the final questionnaire.

### Statistical analysis

Data were analyzed by (SPSS Inc., Chicago, IL, USA). Continuous variables were expressed in the mean and standard deviation or medians with interquartile range (IQR) values, while categorical variables were presented in frequency and percentages. An agreement was assessed at the individual item level for both content validity and test-retest reliability. The Kappa coefficient (K) and percentage agreement (%A) the question items were used to evaluate the test-retest reliability. The items were accepted if they passed at least one of two set criteria [35, 36]; Criterion one: K ≥ 0.61 = good or %A ≥ 90% and Criterion two: K ≥ 0.51 = moderate and % A ≥ 80%. We omitted items with multiple response options and open response option in the Kappa coefficient (K) and percentage agreement (%A) analysis because their analysis requires only one option. The percentage of students who gave the right answer to the item was used to calculate the level of item difficulty and used the Pearson's correlations coefficients. The p values < 0.05 level were considered as statistically significant for all analyses and specifying one-tailed test of significance.

### Ethics and research approval

The institutional approval for the study was obtained from the ethics and research committee of the University of Ilorin Teaching Hospital (ERC Protocol No–PAN/2017/03/1649). A written form of consent was obtained from the doctors and nurses, and the data collected were analyzed anonymously.

## Results

### Questionnaire design

The initial draft of the knowledge of acute oxygen therapy questionnaire (AOTQ) contained 44 questions in 11 sections detailed below:

Section 1: Professional and prior oxygen use-12 questions
Section 2: Sources of oxygen therapy Education-3 questions
Section 3: Awareness of oxygen therapy guideline-3 questions
Section 4: General knowledge of oxygen-5 questions
Section 5: Recognizing hypoxaemia & tissue hypoxia- 5questions
Section 6: Indication for oxygen therapy -1question
Section 7: Documentation for delivery of oxygen-3 questions
Section 8: Oxygen delivery practices- 8 questions
Section 9: Monitoring of oxygen therapy- 1question
Section 10: Weaning and discontinuation of oxygen therapy- 1 question
Section 11: Oxygen toxicity-1 question

### Face validity

All respondents reviewed each of the 44 questions and indicated that they understood the questions. Five of them found them easy to answer, and four of them also suggested that the appearance and layout would be acceptable to the intended target audience. Most of the respondents suggested splitting of item 9 into two because of its ambiguity and also to ensure clarity. The pediatrician and obstetrician recommended expanding the indications for acute oxygen therapy to include obstetrics and pediatric emergency in the next version of the questionnaire. At the end of face validity, the AOTQ has a total of 45 questions consisting of eight questions on professional profiles and the 37 questions on knowledge, awareness and previous oxygen therapy that were sent for content validity.

### Content validity

The panel of expert reviewed the 37 questions on knowledge, awareness and previous oxygen therapy. The revised AOTQ, after a panel of expert review, contained 31 questions with 52 response items. This is because ten questions were expunged from original 37 questions leaving the remaining 27 questions and additional four questions were also created from a question with many options. Out of the ten that were removed, the three questions were considered to be irrelevant and repetitions, six questions were removed because of low agreement among the reviewers, CVI <0.78 and one was deemed to be applicable only to doctors and not to the nurses and was removed despite the acceptable CVI. It was also recommended that questions four question on documentation of oxygen prescription should be redrafted to have three questions, each with a single correct option to ensure clarity and reduce the volume of the instrument. The panel of expert also suggested that question on the indications for oxygen with ten response options should be redrafted into five separate questions with yes and no response for ease of summation of items. This created an additional four questions making a total of 31 questions (Table 1). At the end of content validity, the AOTQ has a total of 39 questions consisting of 31 questions on knowledge, awareness and previous oxygen therapy and eight questions on professional profiles (S1 Appendix)

**Table 1:** Content validity index of questions items.

Questions number	Content Validity Index	Expert Recommendation	Questions number	Content Validity Index	Expert Recommendation
9.	1	F	35.	0.8	F
10.	0.8	F	36	0.9	F
11.	**0.7**	NF	**37.**	**0.7**	NF
12.	**0.7**	NF	38.	0.9	F
13.	*0*.*8*	NF	39	0.9	F
14.	1	F	**40**	**0.7**	NF
15.	1	F	**41**	**0.7**	NF
16.	0.8	F	42.	*0*.*8*	NF
17.	1	F	**43.1**	**0.6**	NF
18.	0.9	F	**43.2**	**0.6**	NF
19.	1	F	**43.3.**	**0.6**	NF
20.	0.9	F	**43.4**	**0.6**	NF
21.	1	F	**43.5**	**0.6**	NF
22.	1	F	**43.6**	**0.6**	NF
23.	1	F	**43.7**	**0.6**	NF
24.	0.9	F	**43.8**	**0.6**	NF
25.	1	F	**43.9**	**0.6**	NF
26.	0.8	F	**43.10**	**0.6**	NF
27.	0.9	F	44.1	0.8	Reduce to 1 out
28.	0.9	F	44.2	0.8	of 3
29.	1	F	44.3	0.8	Answers/Options
30.1	0.8		44.4	0.8	
30.2	0.8		44.5	0.8	
30.3	0.8		**45.0**	**0.7**	
30.4	0.8	Rephrases to 5			
30.5	0.8	MCQs with one			
30.6	0.8	Answers/Options			
30.7	0.8				
30.8	0.8				
30.9	0.8				
30.10	0.8				
31.	0.8	F			
32.	0.8	F			
33.	0.8	F			
**34.**	**0.6**	NF	favourable	28	
			S-CV1	85%	

S-CVI-Global Content validity index is the arithmetic mean of the CVIs. Bold–Removed question when CVI <0.78. Italics- Removed question based on the expert recommendation. Favorable (F) rating- the question item captures the topic, is readable, clear, brief and comprehensive. Not Favorable (NF) rating—the question item does not captures the topic, lacks readability, clarity, brevity and not comprehensive

### Pilot study and questionnaire revision

Sixteen (80%) out of the 20 nurses and doctors invited participated in the pilot study. All the questions were well understood by the participants but suggested a rewording of some questions. For questions with a factual statement, the respondents suggested changing the answers options from yes/no to true or false. Finally, some of the respondents suggested the inclusion of an optional question on the challenges of oxygen administration considering the lack of infrastructures in the limited resource setting and rural communities.

### Test-retest analysis

A total of 140 doctors and nurses were invited and recruited by convenience sample to participate in the reliability test, 121 completed the test and retest questionnaire in 2–4 weeks giving a dropout rate of 13.6%. Of the 121 respondents, 91(75.2%) were doctors, and 31(24.8%) were nurses. The median years of practice after graduation and working in the hospital were 7(3–10) and 2(1–5) years respectively. The demographic and professional profiles are reported in Table 2.

**Table 2:** Professional profile of the respondents.

Characteristics	N	%
**Gender**		
Male	72	59.9
Female	49	40.1
**Department/Ward**		
Family Medicine	3	2.5
Internal medicine	55	45.5
Obstetrics	18	14.9
Pediatrics	16	13.2
Surgery(Including anaesthesia)	24	19.8
Community Medicine	5	4.1
**Profession**		
Doctors	90	75.2
Nurses	30	24.8
**Additional qualification**		
Yes	8	6.6
No	113	93.4
** Years of practice after graduation(median)**	7(3–10)	
**Duration of working in the current hospital(median)**	2(1–5)	
**Current position/ Job designation**		
Specialist consultant	5	4.1
Senior registrar	24	19.8
Registrar	25	20.7
Medical officer	11	9.1
House officer	25	20.7
Nurse	30	24.8
Others	1	0.8

Data presented in frequency and % or as median with Interquartile range

We presented the Kappa coefficient (K) and percentage agreement (%A) for 30 questions on knowledge; awareness and previous oxygen therapy in the test-retest survey. Question 13 was not analyzed for reliability because it had a multiple option response (See Table 3). The test-retest reliability statistics showed a Kappa coefficient of 0.546–0.897 (all p<0.001) and percentage agreement of 80–98.3% indicating high temporal stability in the target population. Overall, 27 out of 30 analyzed questions fulfilled the reliability acceptance criteria. Questions that did not fulfill the acceptance criteria were 20, 31, and 33 however; questions 33 retained because it was vital in oxygen prescription. At the end of reliability analysis, the AOTQ has a total of 37 questions consisting of 29 questions on knowledge; awareness and previous oxygen therapy and eight questions on demographics and professional profiles.

**Table 3:** Reliability analysis of the questions in the test-retest survey.

S/N	Questions number	Questions	N Test	N Retest	Percent Agree ment	Kappa Coeff. (K)	Reliability
1.	Q9	How long ago did you administer oxygen to a patient?	121	121	91.7	0.798	Cr 1
2.	Q10	How long ago did you prescribe oxygen to a patient?	121	121	91.7	0.824	Cr 1
3.	Q11	Aside from the undergraduate or basic professional training, have you received any CME/ update/ special training on oxygen therapy	121	121	93.4	0.762	Cr 1
4.	Q12	What year did you receive the update/training?	24	24	90.9	0.829	Cr 1
5.	Q13	What is your major source of information on oxygen therapy?	-	-	-	-	No Analysis
6.	Q14	Are you aware of any Guideline on Oxygen Therapy	119	121	88.2	0.724	Cr 1
7.	Q15	Have you ever read the guideline?	119	121	92,2	0.732	Cr 1
8.	Q16	Have you ever used/applied the guideline in your practice	118	121	89.6	0.657	Cr 1
9.	Q17	Oxygen is like any other medication	120	121	95.8	0.897	Cr 1
10.	Q18	Oxygen is not medication but a supportive therapy	120	121	88.2	0.745	Cr 1
11.	Q19	Oxygen should only be given after doctors’ prescription	120	121	90.1	0.809	Cr 1
**12.**	**Q20**	**Oxygen may cause harm when used inappropriately**	**121**	**120**	**95.8**	**0.374**	**No Criteria Satisfied**
13.	Q21	Oxygen promotes combustion	118	117	92.2	0.546	Cr 2
14.	Q22	Hypoxaemia can be recognized by clinical signs	120	120	98.3	0.650	Cr 1
15.	Q23	Arterial Blood Gas Analysis(ABG) is useful for confirming hypoxaemia	119	119	95.8	0.601	Cr 2
16.	Q24	Breathlessness is not always a sign of hypoxaemia	118	120	81.2	0.600	Cr 2
17.	Q25	Pulse Oximetry is useful in detecting and monitoring	121	120	98.3	0.714	Cr 1
18.	Q26	SpO2 level < 90% in adults define hypoxaemia	119	120	88.1	0.551	Cr 2
19.	Q27	Indication for Acute Oxygen Therapy Central Cyanosis	119	121	96.6	0.600	Cr 2
20.	Q28	Indication for Acute Oxygen Therapy include Asymptomatic Aneamia	119	119	88.4	0.719	Cr 1
21.	Q29	Indication for Acute Oxygen Therapy include Eclampsia	118	121	92.4	0.719	Cr 1
22.	Q30	Indication for Acute Oxygen Therapy include Restlessness and Convulsion in children	116	118	86.0	0.611	Cr 1
**23.**	**Q31**	**Indication for Acute Oxygen Therapy include Respiratory distress (respiratory rate >24/min in adult or 60 in neonate)**	**119**	**120**	**85.6**	**0.290**	**No Criteria Satisfied**
24.	Q32	Which of the following should be documented in the prescription chart of a patient receiving oxygen	121	120	83.8	0.559	Cr 2
25.	*Q33*	*Which of the following should be documented in the prescription chart of a patient receiving oxygen*	*118*	*119*	*87*.*6*	*0*.*484*	*No Cr but* *Retained*
26.	Q34	Which of the following should be documented in the prescription chart of a patient receiving oxygen	118	119	94.0	0.601	Cr 2
27.	Q35	Which of the following statement on the prescription of oxygen and delivery is true?	110	112	80.6	0.697	Cr 1
28.	Q36	A 72-year-old farmer with COPD has carbon dioxide retention (type II respiratory failure), which of this delivery device is appropriate for oxygen delivery?	112	116	86.6	0.729	Cr 1
29.	Q37	A 12-year-old boy had type 1 respiratory failure, select one correct initial dose of oxygen	112	115	88.3	0.599	Cr 2
30.	Q38	Humidification is essential for patients receiving oxygen through one the following device:	120	121	80.0	0.689	Cr 1
31.	Q39	Regarding weaning and discontinuation of oxygen which of these recommendations is correct	121	120	92.5	0.610	Cr 1

Criterion one (Cr 1): K ≥ 0.61 = good or %A ≥ 90% and Criterion two (Cr 2): K ≥ 0.51 = moderate and % A ≥ 80%. Bold–Removed question when Cr 1 or Cr -2 was not satisfied. *Italics*- Retained question based on the expert recommendation. Q13 –no analysis because of multiple responses

With regards to the question item analysis, three questions (Q 27, 34 and 35); out of the 21 questions on knowledge of acute oxygen therapy had unacceptable item difficulty level and item-to-total correlations (Table 4). The questions were retained because they tested the adequacy of oxygen prescription which is a fundamental concept of acute oxygen therapy. Furthermore, they performed well in other tests like content validity and test-retest reliability.

**Table 4:** Item difficulty and discrimination.

Questions number	Difficulty (% answering correctly)	Discrimination (item-total r value)
Q17	46(38.0)	0.49
Q18	48(39.7)	0.51
Q19	72(59.5)	0.02*
Q20	-	-
Q21	106(87.6)	0.38
Q22	116(95.9)	0.29
Q23	110(90.9)	0.28
Q24	68(56,2)	0.18*
Q25	116(95.9)	0.20
Q26	98(81.0)	0.14*
Q27	111(91.7)	0.12*
Q28	92(76.0)	0.23
Q29	94(77.7)	0.23
Q30	78(64.5)	0.23
Q31	-	-
Q32	88(72.7)	0.31
*Q33*	108(89.3)	0.33
Q34	106(87.6)	0.18*
Q35	23(19.0)	0.03*
Q36	72(59.5)	0.27
Q37	94(77.7)	0.37
Q38	37(30.6)	0.23
Q39	109(90.1)	0.28

* *p*-values Not significant

Q20 & Q31 removed because of poor reliability

The final draft has eight sections and 37 questions made up of 8 demographic and professional profiles, 29 questions on knowledge; awareness and previous oxygen therapy. An optional open-ended question on challenges of oxygen administration was included in the questionnaire based on the suggestion of the piloted participants (S2 Appendix).

## Discussion

This study showed that the global content validity index (CVI) of the questionnaire (S-CVI) was 0.85, higher than the value defined as acceptable according to the criteria established in the scientific literature [29]. The content validity index used in this study was not able to distinguish chance agreement unlike the Kappa coefficient, it is imperative to point out that a dichotomous scale was used in some question items that do not allow for chance agreement among panelists. This property of content validity index (CVI) makes it a very robust measure of content validity and hence eliminates ambivalence and allows straightforward interpretation [30].

The reliability test and decision to accept a question with its item in the AOTQ was based on the percentage agreement and the Kappa coefficient calculated. One drawback of the use of Kappa for stability measurement is that it does not take into account the degree of disagreement [36]. We, therefore, complemented the Kappa with percentage agreement in the questions when K is low to help in the stability acceptance decision [35–36]. This has helped in deciding for questions 21, 23, 24, 26, 27, 32, 34 and 37 (low K and high percentage). Questions 20, 31, 33 were not stable, but question 33 was retained because of the importance of documentation of delivery device in oxygen prescription. Thus two questions were removed from the final draft of the AOTQ. In this study, the most stable question was about oxygen as any other medication, this was not situation-dependent, and the answer is therefore expected to reflect the general knowledge of medical oxygen (example question 17). However, questions number 31 was the least stable because it is situation-dependent and is a function of the patient physiology and other comorbid conditions.

Most questions were at an appropriate level (item-to-total correlations >0.2). Three out of the 29 questions about knowledge of acute oxygen therapy had low item discrimination. Q27 was testing central cyanosis as an indication for oxygen; Q34 and Q35 were testing required documentation in oxygen prescription. All the three questions were retained because they were also testing an important area of oxygen therapy for proper prescription and administration that are in line with international best practices [2, 7, 14, 15, 17].

The findings from our study supported AOTQ as a valid instrument to measure doctors and nurses level of knowledge of acute oxygen therapy in a simple and efficient way because of its brevity and capability for self-administration. There is a huge gap in the knowledge of health care providers on various aspects of oxygen therapy, and this knowledge deficiency may also be a barrier to optimal oxygen administration [1]. The AOTQ can be a useful tool in the university and hospital to improve the knowledge about oxygen therapy of Doctors, Nurses, medical and nursing students. The AOTQ can be used as an exploratory tool to gather baseline information and in-depth knowledge about acute oxygen administration among doctors and nurses before the introduction of oxygen prescribing protocol or guideline. The baseline data can be compared with subsequent, post-intervention surveys. Also, the AOTQ can be a useful tool in future studies for assessing knowledge gaps and facilitating a better understanding of barriers to oxygen therapy guidelines. Furthermore, it can be utilized to design intervention and test the acceptability of an educational intervention programme, set a priority for specific areas of knowledge deficiency. Besides, the information gotten can enrich and inform oxygen prescribing policy/guideline champions with a credible basis for strategic decision-making.

The strength of this study is the rigorous methodology employed in the development of this questionnaire which included face validity, the incorporation of an expert panel to assess the content validity and test-retest reliability. The significant limitations of this study are that it was conducted only among hospital-based doctors and nurses and the result could not be generalized to all categories of physician and nurses. The questionnaire is only available in the English language, and we do not know how well this questionnaire will perform in other languages. This AOTQ was developed, piloted and validated in the Nigerian health care system. In other healthcare settings, with different working conditions, there may be a need for validity and stability testing of the AOTQ. Additional limitations were the moderate sample size of respondents and the convenience sampling method of recruitment of respondents.

In conclusion, this questionnaire is a reliable and valid tool for assessing knowledge of acute oxygen therapy among doctors and nurses. This questionnaire will bridge the gaps of unavailability of reliable and validated instruments to gather information about the oxygen therapy. It will also aid a better understanding of oxygen treatment and help healthcare policymakers to formulate educational intervention workshops to improve oxygen administration practices.

## Supporting information

S1 Appendix Revised AOTQ after content validity. The correct responses are in blue color.
(DOC)

S2 Appendix Final AOTQ. (DOC)

S3 Appendix Data file. (ZIP)

## References

[1] Cousins J, Wark P, McDonald V. Acute oxygen therapy: a review of prescribing and delivery practices. International Journal of Chronic Obstructive Pulmonary Disease. 2016; 11(1): 1067–1075. 10.2147/COPD.S103607 27307722 PMC4888716

[2] Bateman NT, Leach RM. ABC of oxygen: acute oxygen therapy.BMJ. 1998; 317:798–801 9740573 10.1136/bmj.317.7161.798 PMC1113909

[3] Martin DS, Grocott MP III. Oxygen therapy in anesthesia: the yin and yang of O2. Br J Anaesth. 2013; 111(6):867–871. 10.1093/bja/aet291 24233308

[4] Cabello JB, Burls A, Emparanza JI, Bayliss S, Quinn T. Oxygen therapy for acute myocardial infarction. Cochrane Database Syst Rev 2010; 6:CD007160. 10.1002/14651858.CD007160.pub2 20556775

[5] Wijesinghe M, Perrin K, Ranchord A, Simmonds M, Weatherall M, Beasley R. Routine use of oxygen in the treatment of myocardial infarction: systematic review. Heart 2009; 95:198–202. 10.1136/hrt.2008.148742 18708420

[6] Ronning OM, Guldvog B. Should stroke victims routinely receives supplemental oxygen? A quasi-randomized controlled trial. Stroke 1999; 30:2033–2037. 10512903 10.1161/01.str.30.10.2033

[7] O’Driscoll BR, Howard LS, Davison AG; British Thoracic Society. BTS guideline for Emergency oxygen use in adult patients. Thorax 2008; 63(Suppl 6):vi1e68. 18838559 10.1136/thx.2008.102947

[8] Joosten SA, Koh MS, Bu X, Smallwood D, Irving LB. The effects of oxygen therapy in patients presenting to an emergency department with exacerbation of chronic obstructive pulmonary disease. Med J Aust. 2007; 186(5):235–238 17391084 10.5694/j.1326-5377.2007.tb00879.x

[9] Austin MA, Willis KE, Blizzard L, Walters EH, Wood-Baker R. Effect of high flow oxygen on mortality in chronic obstructive pulmonary disease patients in prehospital setting: randomized controlled trial. BMJ. 2010; 341(c5462). 10.1136/bmj.c5462 PMC2957540 20959284

[10] Wijesinghe M, Perrin K, Healy B, Hart K, Clay J, Weatherall M et al Pre-hospital oxygen therapy in acute exacerbations of the chronic obstructive pulmonary disease. Intern Med J. 2011; 41(8):618–622. 10.1111/j.1445-5994.2010.02207.x 20214690

[11] Abdo WF, Heunks LMA. Oxygen-induced hypercapnia in COPD: myths and facts. Critical Care. 2012; 16(323):4. 10.1186/cc11475 PMC3682248 23106947

[12] Aubier M, Murciano D, Milic-Emili J, Touaty E, Daghfous J, Pariente R, et al: Effects of the administration of O2 on ventilation and blood gases in patients with the chronic obstructive pulmonary disease during acute respiratory failure. Am Rev Respir Dis. 1980, 122: 747–754. 10.1164/arrd.1980.122.5.747 6778278

[13] Global Initiative for Chronic Obstructive Lung Disease (GOLD). Global Strategy for the Diagnosis, management, and Prevention of Chronic Obstructive Pulmonary Disease [updated 2016]; 2016: i–111.

[14] Beasley R, Chien J, Douglas J, Eastlake L, Farah C, King G et al Thoracic Society of Australia and New Zealand oxygen guidelines for acute oxygen use in adults: ‘Swimming between the flags.' Respirology. 2015; 20(8):1182–1191. 10.1111/resp.12620 26486092 PMC4654337

[15] O'Driscoll BR, Howard L, Paris J, Mak V; BTS Emergency Oxygen Guideline Group. BTS Guidelines for oxygen use in adults in healthcare and emergency settings [Public consultation draft]. 2015. Available from: https://www.brit-thoracic.org.uk/document-library/clinical-information/oxygen/emergency-oxygen-guideline-2015/bts-full-guideline-for-oxygen-use-in-adults-in-healthcare-and-emergency-settings-2015/

[16] Lamont T, Luettel D, Scarpello J, O’Driscoll BR, Connew S. Improving the safety of oxygen therapy in hospitals: summary of a safety report from the National Patient Safety Agency. BMJ. 2010; 340:313–314. 10.1136/bmj.c187 20103511

[17] World Health Organization 2011. Ed Trevor D. The clinical use of oxygen in hospitals with limited resources: Guidelines for health-care workers, hospital engineers, and managers

[18] Wijesinghe M, Shirtcliffe P, Perrin K, Healy B, James K, Weatherall M et al An audit of the effect of oxygen prescription charts on clinical practice. Postgrad Med J. 2010; 86(1012):89–93. 10.1136/pgmj.2009.087528 20145057

[19] Gunathilake R, Lowe D, Wills J, Knight A, Braude P. Implementation of a multicomponent intervention to optimize patient safety through improved oxygen prescription in a rural hospital. Aust J Rural Health. 2014; 22(6):328–333. 10.1111/ajr.12115 25495628

[20] Holbourn A, Wong J. Oxygen prescribing practice at Waikato Hospital does not meet guideline recommendations. Intern Med J. 2014; 44(12a):1231–1234. 10.1111/imj.12602 25316385

[21] Boyle M, Wong J. Prescribing oxygen therapy. An audit of oxygen prescribing practices on medical wards at North Shore Hospital, Auckland, New Zealand. N Z Med J. 2006; 119(1238): U2080 16868577

[22] Bell C. Is this what the doctor ordered? Accuracy of oxygen therapy prescribed in hospital. Prof Nurse. 1995; 10(5):279–300. 7708785

[23] Asciak R, Fenech VA, Gatt J, Montefort S. Oxygen prescription and administration at the emergency department and medical wards in Mater Dei Hospital. Malta Med J. 2011; 23(2):19–23.

[24] Dodd ME, Kellet F, Davis A, Simpson JCG, Webb AK, Haworth CS, et al Audit of oxygen prescribing before and after the introduction of a prescribing chart. BMJ. 2000; 321:864–5. 11021863 10.1136/bmj.321.7265.864 PMC27494

[25] Wong C, Visram F, Cook D, Griffith L, Randall J, O'Brien B, et al Development, dissemination, implementation and evaluation of a clinical pathway for oxygen therapy. CMAJ. 2000; 162:29–33. 11216195 PMC1232226

[26] Neves JT, Lobão MJ; Grupo de trabalho EMO. An oxygen therapy multicentric study–a nationwide audit of oxygen therapy procedures in internal medicine wards. Rev Port Pneumol. 2012; 18(2):80–85. 10.1016/j.rppneu.2012.01.001 22280829

[27] DeVon HA, Block ME, Moyle‑Wright P, Ernst DM, Hayden SJ, Lazzara DJ, et al A psychometric toolbox for testing validity and reliability. J Nurs Scholarsh 2007;39:155‑64 10.1111/j.1547-5069.2007.00161.x 17535316

[28] Bolarinwa OA. Principles and methods of validity and reliability testing of questionnaires used in social and health science researches. Niger Postgrad Med J 2015; 22:195–201. 10.4103/1117-1936.173959 26776330

[29] Polit DF, Beck CT. The content validity index: Are you sure you know what’s being reported? Critique and recommendations. Res Nurs Health 2006; 29:489‑97. 10.1002/nur.20147 16977646

[30] Sangoseni O, Hellman M, Hill C. Development and validation of a questionnaire to assess the effect of online learning on behaviors, attitude and clinical practices of physical therapists in the United States regarding of evidence‑based practice. Internet J Allied Health Sci Pract 2013;11:1‑12

[31] Vargas D, Luis MA. Development and validation of a scale of attitudes towards alcohol, alcoholism, and alcoholics. Rev Lat Am Enfermagem. 2008; 16(5):895–902. 19061028 10.1590/s0104-11692008000500016

[32] Marx RG, Menezes A, Horovitz L, Jones EC, Warren RF. A comparison of two-time intervals for test-retest reliability of health status instruments. J Clin Epidemiol 2003; 56(8):730–735 12954464 10.1016/s0895-4356(03)00084-2

[33] Lord FM. The relationship of the reliability of multiple-choice test to the distribution of item difficulties. Psychometrika 1952;18:181–194.

[34] Streiner DL, Norman GR. Health Measurement Scales: a Practical Guide to Their Development and Use. New York: Oxford University Press, 1995

[35] Polit D.F.; Beck C. Essentials of Nursing Research, 6th ed.; Lippincott Williams: Philadelphia, PA, USA, 2006.

[36] Altman DG: Practical Statistics for Medical Research London: Chapman & Hall;1991

