# Supplementary material for: Development and validation of a questionnaire to assess the doctors and nurses knowledge of acute oxygen therapy
Source: PLoS One. 2019 Feb 4;14(2):e0211198. doi: 10.1371/journal.pone.0211198 (PMC6361442; doi:10.1371/journal.pone.0211198)
Supplement: S1 Appendix — The correct responses are in blue color. (DOC) [file pone.0211198.s001.doc]

**Development and Validation of Knowledge of Acute Oxygen Therapy Questionnaire**

Study no………… Test ………… Retest……………….

**SECTION A: GENERAL CHARACTERISTICS**

1. Age (at last birthday in years)..............................................
2. Sex Male Female
3. Department…………………………… Unit/Ward (if applicable) ……………
4. What is your profession? Doctor [] Nurse [] other [] specify…………
5. Additional qualification aside MBBS/ChB/RN /SRM (Specify please) ….………………
6. Years of practice after graduation…………………….
7. Current position/Job Designation in your profession…………………………
8. How long have you been working in this hospital or health facility? …………………
9. How long ago did you administer oxygen to a patient? <1month ago []>1month ago [] >6 month ago[]
10. How long ago did you prescribe oxygen to a patient? <1month ago [] >1month ago [] > 6month ago[]

**SECTION B: RELEVANT EDUCATIONAL BACKGROUND**

1. Aside from the undergraduate or basic professional training, have you received any CME/ update/ special training on oxygen therapy? Yes[] No []
2. If yes to question 11, what year did you receive the update/training?...............
3. What is your major source of information on the oxygen therapy? (Tick one or more please)
4. Medical/Nursing training
5. Post qualification /in-service training
6. Colleagues
7. Journals
8. Print & electronic media
9. Others (specify)……………………………
10. Are you aware of WHO / British/any other guideline on Oxygen Therapy Yes[] No []
11. Have you ever read it? Yes[] No []
12. Have you ever used or applied it your practice Yes[] No [] 13

**SECTION C: KNOWLEDGE OF OXYGEN.**

1. Oxygen is like any other medication True[] False[]
2. Oxygen is not medication but a supportive therapy True[] False[]
3. Oxygen should only be given after doctors’ prescription True[] False[]
4. Oxygen may cause harm when used inappropriately True[] False[]
5. Oxygen promotes combustion True[] False[]

**SECTION D: RECOGNISING HYPOXAEMIA**

1. Hypoxaemia can be recognized by clinical signs True[] False[]
2. Blood Gas Analysis is useful for confirming hypoxaemia True[] False[]
3. Breathlessness is not always a sign of hypoxaemia True[] False[]
4. Pulse Oximetry is a useful in detecting and monitoring hypoxaemia True[] False[]
5. SpO2 level < 90 % in adults define hypoxaemia True[] False[]

**SECTION E: INDICATION FOR ACUTE OXYGEN THERAPY**

Indication for Acute Oxygen Therapy include

1. Central Cyanosis True[] False[]
2. Asymptomatic Anaemia True[] False[]
3. Eclampsia True[] False[]
4. Restlessness and Convulsion in children True[] False[]
5. Respiratory distress (respiratory rate >24/min in adult or 60 in neonate ) True[] False[]

**SECTION F: DOCUMENTATION FOR DELIVERY OF OXYGEN**

1. Which of the following should be documented in the Treatment/Monitoring Chart of a patient receiving oxygen?
2. Oxygen Volume
3. Oxygen Flow Rate or FIO2
4. Oxygen Diffusion Rate
5. Which of the following should be documented in the Treatment/Monitoring Chart of a patient receiving oxygen?
6. Oxygen Solubility
7. Oxygen Source and Delivery Device
8. Oxygen Density
9. Which of the following should be documented in the Treatment/Monitoring Chart of a patient receiving oxygen?
10. Oxygen Odour
11. Frequency of Administration
12. Oxygen and Nitrogen Concentration

**SECTION G: OXYGEN DELIVERY PRACTICES**

1. Which one of the following oxygen delivery device matches the appropriate statement?
2. Nasal catheter oxygen flow rate >5L/min lead to rebreathing of CO2
3. Oxygen prescription should be to a target saturation range rather than a fixed dose
4. Oxygen concentrator delivers maximum oxygen concentration of 70%
5. A 72-year-old farmer with COPD has carbon dioxide retention (type II respiratory failure), which of this delivery device is appropriate for oxygen delivery achieve a target saturation of 88-92%?
6. Nasal catheter at 1-2 L/min/ in the absence of Venturi masks
7. Nasal catheter at 16 L/min
8. Oxygen mask with reservoir 6-9L/min
9. 12-year-old boy had type 1 respiratory failure, select one correct initial dose of oxygen to achieve a target saturation of 94-98%.
10. FiO2 of 60%
11. FiO2 of 20%
12. FiO2 of 150%
13. Humidification is essential for patients receiving oxygen through one the following:
14. Endotracheal tube or a tracheostomy
15. Nasal Prong
16. Oxygen mask
17. Regarding weaning and discontinuation of oxygen which of the following statement is correct?
18. Weaning and discontinuation of oxygen therapy should be started if clinically stable on low-dose oxygen
19. Weaning and discontinuation of oxygen therapy should be started after a new Chest Radiograph is normal
20. Weaning of oxygen therapy should be started if clinically stable on high-dose oxygen

31
