# Supplementary material for: Development and validation of a questionnaire to assess the doctors and nurses knowledge of acute oxygen therapy
Source: PLoS One. 2019 Feb 4;14(2):e0211198. doi: 10.1371/journal.pone.0211198 (PMC6361442; doi:10.1371/journal.pone.0211198)
Supplement: S2 Appendix — (DOC) [file pone.0211198.s002.doc]

**Assessment of Doctors’ and Nurses’ Knowledge of Acute Oxygen Therapy**

**INFORMATION SHEET**

**PURPOSE OF THE STUDY**

The purpose of the study is primarily to determine knowledge of oxygen therapy in among doctors’ and nurses’ in Ilorin, Nigeria. This study will enable the researchers to obtain a baseline data in formulating oxygen protocols for the hospital.

**WHAT IS EXPECTED OF YOU IF YOU AGREE TO PARTICIPATE**

It is expected that those who agree to participate will fill a questionnaire on knowledge of oxygen therapy for 10-15 minutes. The cost of investigations would be borne solely by the researchers and at NO TIME would the patients be required to pay for any tests involved in this study.

**YOUR PARTICIPATION IS VOLUNTARY**- Your participation is voluntary, and you may withdraw at any phase of the study.

**CONFIDENTIALITY** -We will treat information collected from you in absolute confidence. No part or whole of such information shall be divulged to anybody except the investigators. We owe it a duty to keep your records secret.

**BENEFIT OF PARTICIPATION**

Your participation from this study will contribute to clinical practice improvement and patient care. I at this moment consent to participate in the above-stated study as explained to me and I am also aware that I have the right to withdraw my participation at any point during the study if I so wish. Signature Date .

**Please select one answer the by ticking the box and filling the line.**

**SECTION 1: DOCTOR AND NURSE CHARACTERISTICS**

1. How old are you (at last birthday in years)?
2. What is your gender? Male Female
3. Department where you currently work Unit/Ward (if applicable)
4. What is your profession? Doctor Nurse Other
5. Do you have additional qualification aside MBBS/RN? Yes No (If Yes Specify) .
6. How many years have you practice after graduation? .
7. What is your current position/Job designation? .
8. How long have you been working in this hospital or health facility? .
9. How long ago did you administer oxygen to a patient? <1month 1-6 months >6 months
10. How long ago did you prescribe oxygen to a patient? <1month 1-6 months >6 months

**SECTION 2: RELEVANT EDUCATIONAL BACKGROUND**

1. Aside from the undergraduate or basic professional training, have you received any CME/ update/ special training on oxygen therapy? Yes No
2. If yes to question 11, what year did you receive the update/training? .
3. What are your major sources of information on the oxygen therapy? (circle response please)
4. Medical/Nursing training
5. Post qualification /in-service training
6. Colleagues
7. Journals
8. Print & electronic media
9. Others (specify) .

**SECTION 3: AWARENESS AND USE OF OXYGEN THERAPY GUIDELINE**

1. Are you aware of WHO / Any other guideline on Oxygen Therapy? Yes No
2. Have you ever read it? Yes No
3. Have you ever used or applied it your practice? Yes No

**SECTION 4: GENERAL KNOWLEDGE OF MEDICAL OXYGEN.**

1. Oxygen is like any other medication True False
2. Oxygen is not medication but a supportive therapy True False
3. Oxygen should only be given after doctors’ prescription True False
4. Oxygen promotes combustion True False

**SECTION 5: RECOGNISING HYPOXAEMIA**

1. Hypoxaemia can be recognized by clinical signs True False
2. Blood Gas Analysis is useful for confirming hypoxaemia True False
3. Breathlessness is not always a sign of hypoxaemia True False
4. Pulse Oximetry is useful in detecting and monitoring hypoxaemia True False
5. SpO2 level < 90 % in adults define hypoxaemia True False

**SECTION 6: INDICATIONS FOR ACUTE OXYGEN**

Indications for Acute Oxygen Therapy include

1. Central Cyanosis True False
2. Asymptomatic Anaemia True False
3. Eclampsia True False
4. Restlessness and Convulsion in children True False

**Please select one answer by circling either letter a, b, or c**

**SECTION 7: OXYGEN PRESCRIPTION**

1. Which of the following should be documented in the treatment (prescription) chart of a patient receiving oxygen?
2. Oxygen Volume
3. Oxygen Flow Rate or FIO2
4. Oxygen Diffusion Rate
5. Which of the following should be documented in the treatment (prescription) chart of a patient receiving oxygen?
6. Oxygen Solubility
7. Oxygen Source and Delivery Device
8. Oxygen Density
9. Which of the following should be documented in the treatment (prescription) chart of a patient receiving oxygen?
10. Oxygen Odour
11. Frequency of Administration
12. Oxygen and Nitrogen Concentration
13. Which of the following statement on the prescription of oxygen and delivery is correct?
14. Nasal catheter oxygen flow rate >5L/min lead to rebreathing of CO2
15. Oxygen prescription should be to a target saturation range rather than a fixed dose
16. Oxygen concentrator delivers maximum oxygen concentration of 70%

**SECTION 8: OXYGEN DELIVERY PRACTICES**

1. A 72-year-old farmer with COPD has carbon dioxide retention (type II respiratory failure), which of this device is appropriate for oxygen delivery to achieve a target saturation of 88-92%?
2. Nasal catheter at 1-2 L/min/ in the absence of Venturi masks
3. Nasal catheter at 16 L/min
4. Oxygen mask with reservoir 6-9L/min
5. A 12-year-old boy had type 1 respiratory failure, select one correct initial concentration of oxygen to achieve a target saturation of 94-98%.
6. FiO2 of 60%
7. FiO2 of 20%
8. FiO2 of 150%
9. Humidification is essential for patients receiving oxygen through one the following device:
10. Endotracheal tube or a tracheostomy
11. Nasal Prong
12. Oxygen mask
13. Regarding weaning and discontinuation of oxygen which of the following statement is true?
14. Weaning and discontinuation of oxygen therapy should be started if clinically stable on low-dose oxygen
15. Weaning and discontinuation of oxygen therapy should be commenced after a new chest radiograph is normal
16. Weaning of oxygen therapy should be initiated if clinically stable on high-dose oxygen

What are the major challenges of oxygen administration in the hospital ward and emergency room?

1

2

3

Thank you
